# Assessing neural tuning for object perception in schizophrenia and bipolar disorder with multivariate pattern analysis of fMRI data

**DOI:** 10.1016/j.nicl.2017.08.023

**Published:** 2017-09-01

**Authors:** Eric A. Reavis, Junghee Lee, Jonathan K. Wynn, Stephen A. Engel, Mark S. Cohen, Keith H. Nuechterlein, David C. Glahn, Lori L. Altshuler, Michael F. Green

**Affiliations:** a Semel Institute for Neuroscience and Human Behavior, University of California, Los Angeles, Los Angeles, CA 90024; USA; b Desert Pacific Mental Illness Research, Education, and Clinical Center, Greater Los Angeles Veterans Affairs Healthcare System, Los Angeles, CA 90073, USA; c Department of Psychology, University of Minnesota, Minneapolis, MN 55455; USA; d Department of Psychiatry, Yale University School of Medicine, New Haven, CT 06511, USA

**Keywords:** Schizophrenia, Bipolar disorder, fMRI, Multivariate pattern analysis, Visual perception

## Abstract

**Introduction:**

Deficits in visual perception are well-established in schizophrenia and are linked to abnormal activity in the lateral occipital complex (LOC). Related deficits may exist in bipolar disorder. LOC contains neurons tuned to object features. It is unknown whether neural tuning in LOC or other visual areas is abnormal in patients, contributing to abnormal perception during visual tasks. This study used multivariate pattern analysis (MVPA) to investigate perceptual tuning for objects in schizophrenia and bipolar disorder.

**Methods:**

Fifty schizophrenia participants, 51 bipolar disorder participants, and 47 matched healthy controls completed five functional magnetic resonance imaging (fMRI) runs of a perceptual task in which they viewed pictures of four different objects and an outdoor scene. We performed classification analyses designed to assess the distinctiveness of activity corresponding to perception of each stimulus in LOC (a functionally localized region of interest). We also performed similar classification analyses throughout the brain using a searchlight technique. We compared classification accuracy and patterns of classification errors across groups.

**Results:**

Stimulus classification accuracy was significantly above chance in all groups in LOC and throughout visual cortex. Classification errors were mostly within-category confusions (e.g., misclassifying one chair as another chair). There were no group differences in classification accuracy or patterns of confusion.

**Conclusions:**

The results show for the first time MVPA can be used successfully to classify individual perceptual stimuli in schizophrenia and bipolar disorder. However, the results do not provide evidence of abnormal neural tuning in schizophrenia and bipolar disorder.

## Highlights

• Abnormal visual perception exists in schizophrenia and, likely, bipolar disorder
• Neural processing abnormalities underlying those deficits are not well understood
• We used multivariate analyses of fMRI data to assess patients' visual processing
• We establish for the first time that such methods work well in these patient groups
• The analyses did not show group differences in neural processing of visual stimuli

## 1 Introduction

There is strong evidence that visual perception is abnormal in schizophrenia. People with the disorder exhibit poor performance on tasks involving visual masking, contour integration, motion discrimination, and other tests of perception (Butler et al., 2008, Green et al., 2009a, Green et al., 2012, Javitt, 2009, Javitt and Freedman, 2015). Performance on such tests predicts functional outcomes in schizophrenia, suggesting that visual dysfunction might have important cascading effects (Green et al., 2012). There is also emerging evidence that similar, related perceptual deficits may exist in bipolar disorder, an illness that shares some phenotypic characteristics and genetic risk factors with schizophrenia (Chen et al., 2005, Chkonia et al., 2012, Jahshan et al., 2014).

Converging evidence suggests that abnormalities in the structure of visual cortex exist in schizophrenia and related disorders. Postmortem histological studies have shown reductions in the thickness or volume of visual cortex in schizophrenia (Dorph-Petersen et al., 2007, Selemon et al., 1995). Similarly, a recent in vivo structural MRI study found thinner visual cortex in schizophrenia than in controls, with intermediate cortical thickness in bipolar disorder (Reavis et al., 2017).

The function of visual cortex also appears to be abnormal in schizophrenia. Evidence from fMRI suggests that the receptive fields of neurons in early- and mid-level visual areas (V1, V2, and V4) have weaker inhibitory surrounds in schizophrenia than in controls (Anderson et al., 2017). There is also evidence of dysfunction in higher visual areas during perceptual tasks on which patients show deficits. In particular, the lateral occipital complex (LOC), an object-selective region, shows abnormal activity during visual masking and contour integration tasks (Green et al., 2009b, Silverstein et al., 2015).

A ubiquitous property of perceptually driven neurons is *preferential tuning*. Throughout the visual system, individual neurons respond more or less vigorously to stimuli in their receptive fields depending on the degree to which the features of the stimulus match the preference of the neuron. Neurons in early visual cortex are preferentially tuned to basic perceptual features such as orientation. Thus, a V1 neuron might respond most vigorously to stimuli containing image features at a particular orientation, and gradually less vigorously as the difference between the orientation content of the stimulus and the preferred orientation increases (Hubel and Wiesel, 1959). Similar tuning preferences exist in higher visual areas for more complex stimulus features. For example, converging evidence from human and animal studies suggests that neurons in LOC are tuned to visual objects, responding preferentially to specific object features (DiCarlo et al., 2012). Animal studies show that this type of neural tuning depends on gamma-aminobutyric acid-dependent (GABAergic) inhibitory mechanisms (Isaacson and Scanziani, 2011).

Various seemingly disparate perceptual deficits found in schizophrenia and bipolar disorder could be parsimoniously explained by abnormalities in neural tuning. For example, deficits in contour integration and motion perception could each be related to aberrant neural tuning (in orientation- and motion-tuned cells, respectively). To our knowledge, only one published study has investigated neural tuning in schizophrenia. That study measured orientation tuning psychophysically and found evidence consistent with broadened orientation tuning in early visual cortex, which was linked to reduced GABA in that region (Rokem et al., 2011).

We hypothesized that broadened neural tuning for more complex visual features might also exist in schizophrenia or bipolar disorder. LOC contains object-tuned neurons and activity there is aberrant during various perceptual tasks in schizophrenia. Therefore, we decided to investigate tuning for object stimuli with LOC as a primary region of interest (ROI).

Evidence of perceptual tuning abnormalities in schizophrenia or bipolar disorder could provide new insights into the pathophysiology of the illnesses. The neural mechanisms and properties of tuning are similar across sensory modalities, so identification of tuning deficits in the visual system would suggest the possible presence of tuning deficits in other neural systems. Evidence of abnormal visual tuning would suggest a specific pathophysiological mechanism that might underlie various known visual deficits, which predict functional outcomes in schizophrenia. Thus, improved understanding of visual tuning could lead to innovative treatments in the future, either to ameliorate visual perception deficits specifically or to improve neural tuning more broadly across various neural systems.

In the current study, we used multivariate pattern analysis (MVPA) of fMRI data to investigate tuning for objects in schizophrenia and bipolar disorder. Unlike traditional univariate fMRI analyses, which assess the response of individual voxels in different experimental conditions, MVPA compares *patterns* of activity, spanning many voxels, across experimental conditions (Haxby et al., 2001). In our study, we used machine learning techniques to classify patterns of fMRI activity corresponding to specific visual stimuli: a classification algorithm was trained to distinguish experimental conditions based on patterns of activity among voxels in a selected area of the brain, then tested with unlabeled data held out of the training.

With this approach, classification accuracy depends upon the extent to which particular stimuli reliably evoke a unique pattern of activity in a given region, making it an indirect measure of tuning. Broadly tuned neurons would be expected to respond more similarly to different object images than narrowly tuned neurons would. In turn, these more similar neural responses would produce less distinctive patterns of fMRI activity, which are harder to classify accurately.

We performed two types of classification analyses: an ROI-based analysis of data from LOC, and a whole-brain searchlight analysis, which performs MVPA throughout the brain in a comprehensive set of small, overlapping, spherical regions (Kriegeskorte et al., 2006). While LOC is specifically an object-selective area, and thus the main focus of our analyses, we expected that stimulus classification would also be possible in other visual areas. That is because stimuli containing different object-level features must also contain different low-level features (e.g., orientation and spatial frequency content). These low-level differences are expected to evoke different patterns of activity in visual areas tuned to those features (e.g., early visual cortex). Thus, we expected that classification of the stimuli would be possible not only in LOC but throughout visual cortex, and that classification accuracy in each region would be commensurate to the specificity of neural tuning in that area.

Based on the hypothesis that participants with schizophrenia and bipolar disorder have broader visual tuning than healthy controls, we predicted that multivariate classification of object stimuli in LOC and other areas of visual cortex would be less accurate in patients than controls. We expected that this difference would manifest as a significant effect of group in omnibus tests of significance (ANOVAs). However, because we expected to find impairment in both patient groups, for the main analyses we also performed pairwise tests (*t*-tests) to separately compare each patient group to the control group with maximal statistical power.

## 2 Methods and materials

### 2.1 Participants

Participants came from an NIMH-sponsored study of visual processing in major mental illness. In total, 53 schizophrenia patients, 56 bipolar disorder patients, and 53 healthy controls participated in an MRI scan. However, data from a handful of participants in each group were unusable for a variety of reasons (e.g., missing data, excessive motion, or other factors causing data processing to fail). Those subjects were excluded from the analyses, leaving usable samples of 50 schizophrenia patients, 51 bipolar disorder patients, and 47 healthy controls.

All patient participants were clinically stable outpatients with a DSM-IV diagnosis of either schizophrenia or bipolar disorder. Patient participants were on clinically-determined doses of medication and were tested outside of mood episodes. Patients were recruited from outpatient treatment facilities in the Los Angeles area, University of California, Los Angeles (UCLA) outpatient clinics, and mental health clinics at the Veterans Affairs Greater Los Angeles Healthcare System (GLA). Healthy participants were a matched community sample recruited with internet ads. All recruitment methods and experimental procedures were approved by the Institutional Review Boards of GLA and UCLA. All participants provided written informed consent prior to participation.

Selection criteria for all subjects included: a) age 18–65, b) understanding of spoken English sufficient to comprehend testing procedures, c) no evidence of IQ < 70 or developmental disability based on chart review, d) no medical history of clinically significant neurological disease (e.g., epilepsy), e) no history of serious head injury (i.e., loss of consciousness > 1 h, neuropsychological sequelae, cognitive rehabilitation post-head-injury), f) no sedatives or benzodiazepines within 12 h of testing, g) normal or corrected vision, h) no positive urine toxicology screening on day of assessment, i) no known contraindications for MRI scanning, j) no evidence of substance or alcohol dependence in the past three months or of substance or alcohol abuse in the past month, and k) no history of a mood episode in the past two months.

Selection criteria for patient participants included: a) diagnosis of schizophrenia or bipolar disorder based on the Structured Clinical Interview for DSM-IV Axis I Disorders (SCID-I) (First et al., 1997), and b) clinical stability (i.e., no inpatient hospitalizations for 3 months prior to enrollment, no changes in psychoactive medication in the 4 weeks prior to enrollment). Additional selection criteria for healthy controls included: a) no history of psychotic disorder, bipolar spectrum disorder, or other major mood disorder based on SCID-I interview (First et al., 1997) or of avoidant, paranoid, schizotypal, schizoid, or borderline personality disorders based on the Structured Clinical Interview for DSM-IV Axis II Disorders (SCID-II) (First et al., 1996), and b) no family history of a psychotic disorder or bipolar disorder in first-degree relatives, based on participant report.

All SCID interviewers were trained through the Treatment Unit of the Department of Veterans Affairs VISN 22 Mental Illness Research, Education, and Clinical Center to a minimum κ of 0.75 for key psychotic and mood items (Ventura et al., 1998). When available, medical records and reports from treating clinicians were used to corroborate retrospective self-reported information. Patients' clinical symptoms were characterized with the Brief Psychiatric Rating Scale (BPRS), Young Mania Rating Scale (YMRS), and Hamilton Depression rating scale (HAMD) (Hamilton, 1960, Ventura et al., 1993, Young et al., 1978).

### 2.2 MRI data collection

All MRI data were collected at the UCLA Staglin Center for Cognitive Neuroscience on a 3-Tesla Siemens Tim Trio scanner with a 12-channel head coil (Siemens Medical Solutions; Erlangen, Germany). T1-weighted structural scans were collected using a Magnetization-Prepared Rapid Gradient Echo (MPRAGE) sequence (1.9 s TR, 3.4 ms TE, 9° flip-angle, 1 mm isotropic voxels, 256 × 256 × 160 voxel field of view). Functional scans used a standard Echo-Planar Imaging (EPI) sequence (2.5 s TR, 35 ms TE, 75° flip-angle, 3 × 3mm voxels with a 3.3 mm center-to-center inter-slice distance, 64 × 64 voxel field of view with 38 slices). Task stimuli were presented using MR-compatible VisuaStim goggles (Resonance Technology, Inc.; Los Angeles, Calif.), and participant responses were recorded using an MR-compatible response-box.

A functional localizer for LOC was collected for each participant. During the localizer run (2.5 min), participants viewed images of abstract sculptures and scrambled images of those sculptures in alternating 12.5 s blocks (Malach et al., 1995). They were instructed to press a button each time the block-type changed.

After the functional localizer, participants completed five runs of an object-perception task. Each run lasted 3:20. During the task, participants viewed five images: two different chairs, two different cups, and one outdoor scene. In a shuffled order, two 14-second blocks of each image were presented in each run, plus two 14-second blocks containing only a fixation cross. In each object-stimulus block, the image flashed on for 1 s and off for 0.4 s ten times, appearing in slightly different locations each time (~ 1° visual angle jitter). Occasionally, the image presented was missing a part (e.g., a cup missing a handle). Subjects were asked to press a button each time they detected a missing part.

### 2.3 MRI data analysis

Three software packages were used for MRI data analysis. Structural data were processed in FreeSurfer 5.3.0, which parcellated the cortex into anatomical regions used to constrain ROI definitions (Dale et al., 1999, Fischl et al., 1999). Functional data registration, motion correction, and deconvolution, as well as ROI creation, was performed in FSL (Jenkinson et al., 2012, Smith et al., 2004). MVPA analyses were performed using the CoSMoMVPA toolbox (Oosterhof et al., 2016) in MATLAB (MathWorks; Natick, Mass.).

Skull-stripping and cortical reconstruction of the high-resolution anatomical scans, which includes parcellation of the cortex into anatomical regions based on sulcal topology, was performed using the FreeSurfer recon-all process (Dale et al., 1999, Fischl et al., 1999). Results were inspected visually to verify accuracy.

Functional data was processed with FSL FEAT (Woolrich et al., 2009). At that stage of processing, each run was motion-corrected and registered to the subject's skull-stripped anatomical scan using FLIRT (Jenkinson et al., 2002, Jenkinson and Smith, 2001). This procedure calculated the absolute displacement of the functional images at each TR.

We excluded from further analyses any run where the mean absolute displacement across all TRs was > 1.5 mm. Furthermore, subjects for whom more than two runs were unusable according to this criterion were excluded (this criterion resulted in the exclusion of one subject from each group). Deconvolution of the functional data was performed in FEAT by modeling the response of each voxel to the 5 different stimulus conditions (i.e., image types) in that run block-wise, and calculating parameter estimates for each condition. Nuisance regressors for linear motion (identified with FLIRT) and outlier motion (identified with fsl_motion_outliers) were also included in the deconvolution model. No spatial smoothing was applied in the FEAT processing, as it is typically detrimental for MVPA (Misaki et al., 2013).

Functional localizer data were processed using the same FSL pipeline, modeling the intact and scrambled object conditions. LOC ROIs were defined by selecting a proportion of the voxels within the anatomically-parcellated lateral occipital lobe in each hemisphere. Specifically, the lateral occipital label from the Desikan-Killiany atlas was projected into the native functional space of the localizer scan and used as a mask for voxel selection (Desikan et al., 2006).

Primary and exploratory LOC ROIs were created for use with MVPA. Both were made using the functional localizer data, but different thresholds were applied, yielding ROIs of different sizes. The ROIs were defined based on the difference in response between the intact- and scrambled-object localizer conditions. Using fslmaths, voxels within the lateral occipital mask were ranked according to the amount of difference in activity between the two conditions. Then, ROIs were created by selecting different percentages of the ranked voxels, at ten-percent intervals of the robust range (i.e., the top 10%, top 20%, and so on, up to 100%). The 60%-threshold ROI was used for the primary analyses. Exploratory analyses were also run in the ROIs with higher and lower thresholds, to determine whether MVPA results differed for smaller or larger ROIs.

In CoSMoMVPA, we performed classification analyses separately for each subject on the voxel-wise beta-weights (i.e., FSL parameter estimates) from the object-perception task (Mumford et al., 2012). First, the beta-weights were demeaned to ensure classification could not be driven by a difference in the amount of activity by condition across all voxels (i.e., a simple univariate difference). Second, a linear support vector machine (SVM) was trained on labeled data from all but one run of the task. Third, the trained classifier was presented with unlabeled data from the held-out run. Accuracy and details of classification errors were recorded for the attempted classification of the held-out data. This process was repeated five times, using a different run as the holdout sample each time (leave-one-run-out cross-validation). These cross-validated analyses were performed separately for each parametrically thresholded LOC ROI. The resulting measures were compared across groups and conditions using standard statistics. Effect size confidence intervals were computed for each comparison according to the methods of Smithson (2001). For *t*-tests (Cohen's *d*), effect sizes were calculated at the 95% level, but for *F*-tests (partial η^2^), 90% confidence intervals were calculated, as recommended by Steiger (2004).

We also performed a whole-brain searchlight analysis in CoSMoMVPA (Kriegeskorte et al., 2006). This analysis was performed in the same way as the ROI-based analysis, except that the entire brain was tiled with overlapping 3-voxel-radius spherical ROIs, and MVPA was performed within each of them. This yields a whole-brain map for each subject in which the center voxel of each ROI is labeled according to classification accuracy. Nonparametric, permutation-based statistical analyses of these maps to identify effects within and between groups were performed in FSL Randomise with 5000 permutations (Winkler et al., 2014). For within-group effects, we tested for voxels where classification accuracies were significantly above chance within each group (i.e., a nonparametric test similar to a one-sample *t*-test). For between-group effects, we tested for voxels where there were significant differences in classification accuracy across groups, using omnibus and pairwise comparisons (i.e., nonparametric tests analogous to ANOVAs and *t-*tests, respectively). For all tests, we used a threshold of *p* < 0.05, familywise-error-rate-corrected, with threshold-free cluster enhancement. Two bipolar patients were excluded from the searchlight analyses for technical reasons.

## 3 Results

Table 1 contains demographic and clinical information about the study participants. Age, gender, handedness, and parental education did not differ significantly across the three groups. Expectedly, schizophrenia patients had fewer years of personal education. The two patient groups were well-matched for illness duration and did not differ in terms of BPRS, HAM-D, or YMRS symptom ratings.

**Table 1:** Characterization of participants included in the MVPA analyses.

	SZ patients	BD patients	Controls	Group comparison	
	(*N* = 50)	(*N* = 51)	(*N* = 47)		
	Mean (SD)	Mean (SD)	Mean (SD)	Statistic	*p*
Age	46.26 (11.52)	44.78 (12.41)	46.79 (8.14)	*F*(2145) = 0.45	*p* = 0.64
Illness duration (years)	23.76 (12.52)	23.09 (12.96)		*t*(93) = 0.26	*p* = 0.80
Personal education	12.90 (2.23)	14.16 (2.45)	14.49 (1.78)	*F*(2144) = 7.27	*p* = 0.001
Parental education	12.89 (2.81)	13.97 (3.01)	13.69 (3.11)	*F*(2136) = 1.66	*p* = 0.19
Gender (M/F)	32/18	27/24	22/25	χ^2^(2) = 2.99	*p* = 0.22
Handedness (R/L)	43/7	46/4	40/7	χ^2^(2) = 1.29	*p* = 0.53
BPRS (Total)	40.42 (10.73)	33.98 (6.65)		*t*(98) = 3.60	*p* > 0.001
HAM-D (Total)	6.46 (5.05)	6.67 (4.68)		*t*(99) = − 0.21	*p* = 0.83
YMRS (Total)	4.94 (4.10)	3.61 (4.63)		*t*(99) = 1.53	*p* = 0.13

Stimulus classification using MVPA worked well in all groups. In the primary LOC ROI, classification accuracies were distributed normally and were above chance in all three groups. In controls, the mean accuracy was 43.0% (standard deviation = 14.7%), which was significantly greater than the 20% classification performance that would be expected by chance: *t*(46) = 10.71, *p* < 0.001, Cohen's *d* = 1.56 [95% effect size confidence interval = 1.13, 1.99]. Similarly, in schizophrenia patients, accuracy was 41.3% (15.1%), and in bipolar patients it was 41.2% (12.3%). Accuracies in both these groups were also significantly better than chance: schizophrenia *t*(49) = 9.98, *p* < 0.001, *d* = 1.41 [1.01, 1.80], bipolar disorder *t*(50) = 12.32, *p* < 0.001, *d* = 1.73 [1.29, 2.16]. There was no significant difference in classification accuracy across the three groups, *F*(2145) = 0.27, *p* = 0.77, partial η^2^ = 0.004 [90% effect size confidence interval = 0.00, 0.02], and there were no pairwise differences in classification accuracy between either patient group and controls (schizophrenia vs. controls, *t*(95) = − 0.58, *p* = 0.56, *d* = − 0.12 [− 0.52, 0.28]; bipolar vs. controls, *t*(96) = − 0.68, *p* = 0.50, *d* = 0.14 [− 0.53, 0.26]).

**Fig. 1:**
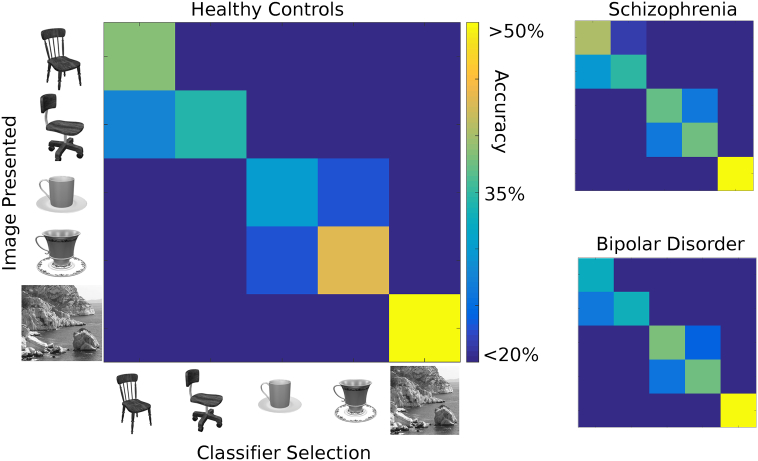
Confusion matrices for each group from the primary LOC ROI. On the y-axis of the table is the type of stimulus presented. On the x-axis is the selection of the trained classifier. Frequencies (in percent) are indicated with the color axis. The figure shows that when classification errors occurred, they were more often within-category than between-category. This effect did not differ by group.

An ANOVA comparing within-category and between-category classification errors for the four types of object stimuli across groups showed an interpretable pattern that did not differ by diagnosis. Fig. 1 shows a confusion matrix indicating the types of classification errors in each group for the primary (60% threshold) LOC ROI. Within-category confusions (e.g., cup vs. cup) were more frequent than between-category confusions (e.g., cup vs. chair) (*F*(1145) = 7.46, *p* = 0.007, partial η^2^ = 0.05 [0.01, 0.12]). However, there was neither a main effect of group (*F*(2145) = 1.07, *p* = 0.35, partial η^2^ = 0.01 [0.00, 0.05]) nor a group-by-error-type interaction (*F*(2145) = 0.85, *p* = 0.43, partial η^2^ = 0.01 [0.00, 0.05]). Pairwise comparisons of the two patient groups and controls showed a similar absence of group main effects (schizophrenia vs. controls, *F*(1,95) = 0.29, *p* = 0.59, partial η^2^ = 0.003 [0.00, 0.05]; bipolar vs. controls, *F*(1,96) = 0.81, *p* = 0.37, partial η^2^ = 0.01 [0.00, 0.06]) or group-by-error-type interaction effects (schizophrenia vs. controls, *F*(1,95) = 0.94, *p* = 0.33, partial η^2^ = 0.01 [0.00, 0.07]; bipolar vs. controls, *F*(1,96) = 0.04, *p* = 0.84, partial η^2^ = 0.0004 [0.00, 0.02]).

**Fig. 2:**
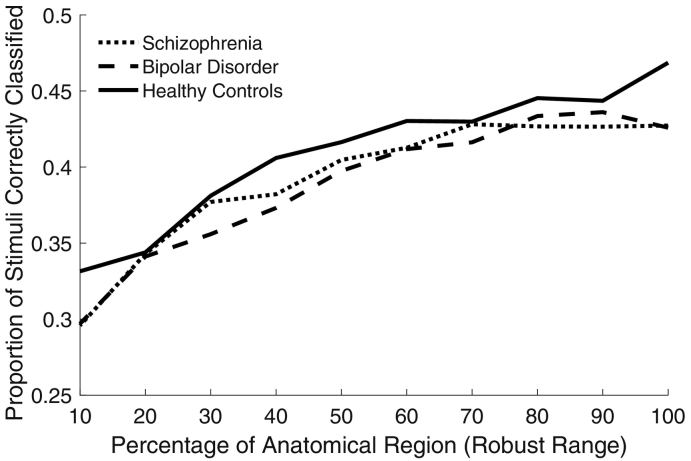
Mean classification accuracy as a function of LOC ROI size, by group. While classification accuracies were higher for larger ROIs, this relationship did not differ significantly across groups.

To evaluate the possible influence of ROI size on the classification accuracy, we varied the size of ROIs parametrically and conducted a repeated measures ANOVA. While there was a main effect of ROI size on classification accuracy (*F*(9,1296) = 49.95, *p* < 0.001, partial η^2^ = 0.26 [0.22, 0.28]) there was neither a main effect of group (*F*(2144) = 0.39, *p* = 0.68, partial η^2^ = 0.01 [0.00, 0.03]) nor a group-by-ROI size interaction (*F*(18,144) = 0.69, *p* = 0.82, partial η^2^ = 0.01 [0.00, 0.04]). Even for the largest ROI (i.e., 100%), where classification accuracies were maximal, there were no differences in classification accuracy across groups (*F*(2145) = 1.12, *p* = 0.33, partial η^2^ = 0.02 [0.00, 0.06]) or between either patient group and controls (schizophrenia vs. controls, *t*(95) = − 1.32, *p* = 0.19, *d* = − 0.27 [− 0.67, 0.13]; bipolar vs. controls, *t*(96) = − 1.30, *p* = 0.20, *d* = − 0.26 [− 0.66, 0.14]). Fig. 2 shows the relationship between ROI size and classification accuracy for each group.

**Fig. 3:**
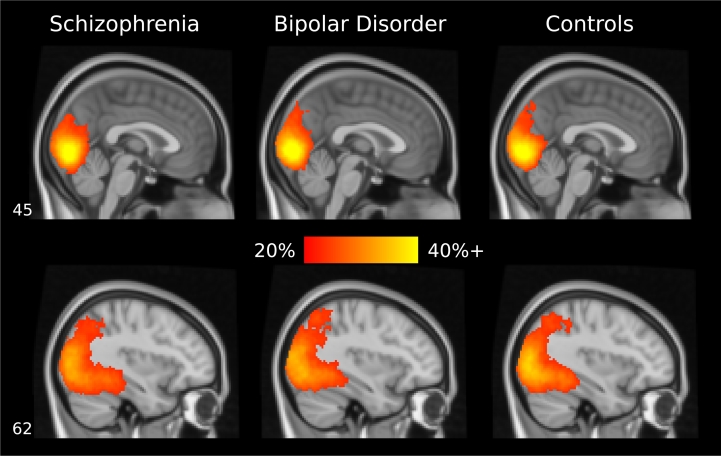
Object-classification searchlight analysis results. Results for each group are displayed on a standard MNI brain. Classification was significantly above chance in the colored voxels (*p* < 0.05, familywise-error-rate-corrected, with threshold-free cluster enhancement). The color axis indicates mean classification accuracy. In all three groups, classification was above chance throughout the occipital lobe, extending slightly into posterior temporal and parietal areas. There were no significant differences in classification accuracy across the three groups anywhere in the brain.

The whole-brain searchlight analysis showed that above-chance classification was possible throughout the occipital lobe and posterior areas of the temporal and parietal lobes, in all three groups. Fig. 3 shows a map of mean classification accuracies for each group, thresholded at *p* < 0.05, familywise-error-rate-corrected, with threshold-free cluster enhancement. The map shows above-chance classification throughout visual cortex in each group, with classification accuracies ranging from slightly above 20% to > 40%. Between-group comparisons of classification accuracy in the searchlight analysis (both omnibus and pairwise) revealed no significant group differences anywhere in the brain, therefore no statistical maps are shown.

To further investigate possible group differences we might have missed, we also performed another type of searchlight analysis as a follow-up. In that analysis, we classified group membership (patient vs. control) based on the data in each searchlight region. We first calculated a condition dissimilarity matrix for each searchlight region, then used the vectorized dissimilarity matrices from a smaller searchlight neighborhood as features in a leave-one-subject-out crossvalidated classification analysis of group membership. This yielded two maps of group membership classification accuracies, one for schizophrenia vs. controls, and one for bipolar vs. controls. To test whether any group classifications were significantly above chance, we performed two Monte Carlo procedures, randomly shuffling the group labels and repeating each subject-classification searchlight analysis 500 times. This generated two null distributions of classification accuracies at each voxel (one for each pair of groups). We then statistically compared the real results at each voxel to the corresponding null distribution using the cosmo_montecarlo_cluster_stat function, which corrects for multiple comparisons and performs threshold-free cluster enhancement. No searchlight regions anywhere in the brain showed above-chance classification of group membership with this approach. Therefore, no statistical maps for this analysis are included among the figures.

## 4 Discussion

We hypothesized that broader tuning for object features in schizophrenia and bipolar disorder would lead to less distinctive patterns of fMRI activity during object perception in patients with those disorders. In turn, we predicted that classification of object stimuli based on multivariate patterns of fMRI activity in LOC and other visual areas would be less accurate in patients than controls. What we found was rather different. Across groups and across multiple analytical approaches, classification of object stimuli was reliably accurate, but we did not detect the group differences we had predicted. The small effect sizes we obtained with relatively large sample sizes suggest that any existing differences between groups are likely to be quite small, and so may be of limited clinical relevance.

MVPA successfully classified patterns of fMRI activity corresponding to perception of specific objects and scenes in schizophrenia, bipolar disorder, and healthy control groups. Classification accuracies were significantly above chance in LOC and throughout visual cortex, in all three groups. Thus, the MVPA approach worked well in patients and controls, but the results of these analyses provide no evidence of imprecise neural tuning in schizophrenia or bipolar disorder.

In LOC, mean classification accuracies were > 40% for all three groups. While this level of classification accuracy might not appear extremely high, it was well above chance performance (20%), and comparable to accuracy levels found in many other fMRI MVPA papers (Mumford et al., 2012). The fact that classification accuracies were above chance shows that the stimuli reliably evoked unique patterns of activity in LOC, and the classification analysis successfully differentiated those patterns.

Beyond the overall classification accuracies, the nature of the classification errors provides further evidence that the method worked well and successfully targeted a correlate of neural tuning for object features. In each group, classification errors in LOC were predominantly within-category (e.g., confusing one chair for another or one cup for another). This suggests that patterns of activity were more similar for similar objects than they were for dissimilar objects.

The LOC results were robust across different thresholds for ROI definition. Parametric analyses of LOC ROIs of different sizes, ranging from inclusion of the entire anatomical area of the lateral occipital lobe to a very conservative functional definition, showed essentially the same result: classification accuracies were significantly above chance in all groups, with no differences in accuracy across groups.

Similarly, a whole-brain searchlight analysis showed that classification accuracy was significantly above chance throughout the occipital lobe, extending slightly into the posterior parietal and temporal lobes, in all three groups. Again, no group differences were found in the searchlight analysis. Thus, each type of stimulus reliably evoked a unique pattern of activity throughout the posterior part of the brain in all three groups. Successful classification throughout visual cortex in the searchlight analysis should not be taken to mean that neurons throughout visual cortex are tuned to objects per se. The stimuli in this study also differed in terms of other visual features (e.g., orientation, spatial frequency), making classification possible in other parts of visual cortex.

Although MVPA techniques have been applied previously in schizophrenia and bipolar disorder, they have been used almost exclusively to classify group membership from brain data (i.e., to try to classify patients vs. controls based on functional or structural neuroimaging data) (Cabral et al., 2016, Koutsouleris et al., 2015, Modinos et al., 2012). This is a very different application of MVPA than our own use of the method to investigate perceptual representations. While our approach is commonplace in cognitive neuroscience, it has seldom been attempted in these psychiatric populations. We are aware of only one other study that used a conceptually similar approach in schizophrenia (Yoon et al., 2008). However, that study used MVPA to classify broad categories of visual stimuli (e.g., faces vs. objects); it did not classify individual stimuli. Thus, to our knowledge this study is the first to perform classification of individual percepts in these clinical populations using MVPA.

Based on our negative finding for group differences in classification, it is possible that neural tuning for visual features is normal in schizophrenia and bipolar disorder. However, there are several reasons that the methods of this study could have missed group differences in neural tuning for visual features. The stimulus set included an extremely small set of common objects (chairs and cups). If tuning deficits exist, it is possible that they are more pronounced for some categories of objects than others. A much larger stimulus set would be required to evaluate this possibility. Thus, the very limited set of stimuli tested in this study is a significant limitation. Also, MVPA might not be the most powerful or direct technique for the assessment of neural tuning; other techniques might be able to target tuning differences more precisely. For example, MR-adaptation techniques use perceptual adaptation to target specific neural populations tuned to objects (or other categories) and measure changes in fMRI activity associated with the adaptation.

Nevertheless, the present results show the utility and strength of the MVPA method in schizophrenia and bipolar disorder. The results show, for the first time, that MVPA can be used to classify patterns of fMRI activity evoked by specific visual stimuli in these patient groups. Indeed, such classification works quite well, not only in LOC, but throughout visual cortex. Our demonstration that MVPA can be used successfully in schizophrenia and bipolar disorder opens the door to broader application of this technique to the study of perception in these disorders. For example, MVPA could be used to analyze fMRI data from tasks in which patients show consistent impairment (e.g., visual masking, contour integration, and biological motion perception). This analytical approach could yield new insights into abnormal brain activity during patients' performance of such tasks. Thus, while the present results are consistent with normal neural activity in schizophrenia and bipolar disorder during a simple object-perception task, they pave the way to sophisticated future investigations of perceptual deficits in these disorders.

## Funding sources and acknowledgments

This work was supported by the National Institutes of Health R01MH095878, to MFG, “Visual Tuning and Performance in Schizophrenia and Bipolar Disorder.” EAR was also supported by the NIH under Ruth L. Kirschstein National Research Service Award F32MH108317. NIMH had no role in the conduct of the study. The authors wish to thank Ana Ceci Meyers, Julio Iglesias, and the rest of the laboratory staff for their help with data collection. This work used computational and storage services associated with the Hoffman2 Shared Cluster provided by UCLA Institute for Digital Research and Education's Research Technology Group.
